# Optimal Scheduling of Campus Microgrid Considering the Electric Vehicle Integration in Smart Grid

**DOI:** 10.3390/s21217133

**Published:** 2021-10-27

**Authors:** Tehreem Nasir, Safdar Raza, Muhammad Abrar, Hafiz Abdul Muqeet, Harun Jamil, Faiza Qayyum, Omar Cheikhrouhou, Fawaz Alassery, Habib Hamam

**Affiliations:** 1 Department of Electrical Engineering, NFC Institute of Engineering and Technology, Multan 60000, Pakistan; 2k18mele3@nfciet.edu.pk (T.N.); safdar.raza@nfciet.edu.pk (S.R.); 2 Department of Electrical Engineering, Bahauddin Zakariya University, Multan 60000, Pakistan; mabrar@bzu.edu.pk; 3 Department of Electrical Engineering Technology, Punjab Tianjin University of Technology, Lahore 54770, Pakistan; abdul.muqeet@ptut.edu.pk; 4 Department of Electronic Engineering, Jeju National University, Jejusi 63243, Korea; 5 Department of Computer Engineering, Jeju National University, Jejusi 63243, Korea; faizaqayyum@jejunu.ac.kr; 6 CES Laboratory, National School of Engineers of Sfax, University of Sfax, Sfax 3038, Tunisia; 7 Department of Computer Engineering, College of Computers and Information Technology, Taif University, Taif 21944, Saudi Arabia; falasser@tu.edu.sa; 8 Faculty of Engineering, Moncton University, Moncton, NB E1A3E9, Canada; Habib.Hamam@umoncton.ca

**Keywords:** distributed generation, distributed energy resources, microgrid, energy management, renewable energy, time of use tariff

## Abstract

High energy consumption, rising environmental concerns and depleting fossil fuels demand an increase in clean energy production. The enhanced resiliency, efficiency and reliability offered by microgrids with distributed energy resources (DERs) have shown to be a promising alternative to the conventional grid system. Large-sized commercial customers like institutional complexes have put significant efforts to promote sustainability by establishing renewable energy systems at university campuses. This paper proposes the integration of a photovoltaic (PV) system, energy storage system (*ESS*) and electric vehicles (EV) at a University campus. An optimal energy management system (EMS) is proposed to optimally dispatch the energy from available energy resources. The problem is mapped in a Linear optimization problem and simulations are carried out in MATLAB. Simulation results showed that the proposed EMS ensures the continuous power supply and decreases the energy consumption cost by nearly 45%. The impact of EV as a storage tool is also observed. EVs acting as a source of energy reduced the energy cost by 45.58% and as a load by 19.33%. The impact on the cost for continuous power supply in case of a power outage is also analyzed.

## 1. Introduction

In this technologically advanced period, the rising demand of energy is fulfilled by fossil fuels that add to most of the greenhouse gas emissions and are becoming expensive and scarce due to climatic changes. Countries around the globe are switching to renewable energy resources (RERs) for energy generation.

RERs are becoming increasingly common due to numerous related benefits such as low carbon emission, no reliance on fuel, clean energy, etc. Regardless of such incredible characteristics, RERs are inherently intermittent and unpredictable as they are weather dependent, resulting in unstable output power. For this reason, energy storage systems are used. Energy storage has piqued a lot of interest globally and will continue to do so in the future [1].

The concept of microgrids has been introduced to enable the integration of RERs evading the need of complex algorithms for managing the renewables. Microgrids are compact energy networks comprising of various loads, conventional and/or renewable distributed energy resources, and energy storage systems [2]. Conventional resources may include several diesel generators and renewable resources that primarily include photovoltaic and wind power systems. A distribution network may contain many microgrids. These microgrids having distributed generators (DGs), *ESS*, and demand response (DR) programs [3], can help lower the electricity cost and load on distribution networks.

Global demand for energy has grown rapidly since 1965 with an average annual rate of 2.2 percent. A growth of 30 percent is expected by 2040 according to world energy outlook. South Asia, along with several other regions in the world, is a significant contributor. It includes eight countries and accounts for 6.42 percent of the overall world energy consumption [4]. Pakistan is one of the developing countries in South Asia that is facing a severe energy crisis ascribed to an ever-increasing population density. It has the sixth largest population in the world [5]. Consumers are subjected to power interruption lasting several hours daily [6]. However, it is an exceptionally rich country in terms of RERs. These resources, if used efficiently, can ultimately mitigate the severe energy crisis. Microgrid implementation is the only viable, safe and optimal approach for ensuring access to cost-efficient, secure, and sustainable energy [7], for all.

The total installed generation capacity of Pakistan is about 38,719 MW, as of June 2020, out of which 24,817 MW is thermal, 9861 MW is hydroelectric, 1248 MW is wind, 530 MW is solar, 369 MW is bagasse, 1467 MW is nuclear, and 427 MW is SPPs/CPPs. The electricity generation has decreased from 136,532 GWh during 2018–2019 to 134,745.70 GWh during 2019–2020, indicating a drop of 1786.30 GWh [8].

**Figure 1:**
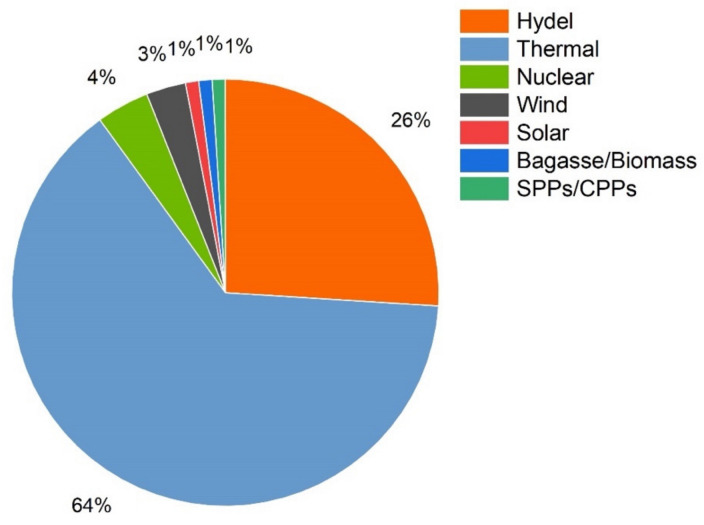
Energy generation sources in Pakistan.

Energy generation shares from different sources in Pakistan are depicted in the chart shown in Figure 1 [9].

Among the various types of heavy load microgrids, institutional buildings fall under the commercial customer’s class. Institutional buildings constitute a diverse variety of load coming from lecture halls, laboratories, libraries, faculty offices, and residential blocks. With onsite distributed generation sources, the prosumers can sell excess power to the grid and can buy power from the grid in case of insufficient generation to meet the load demand. Microgrids taking part in grid functions can reduce loads on power distribution networks.

As power uncertainty is a major issue in the energy sector, institutional microgrids require implementation of optimal energy management systems (EMS) for uninterrupted power supply. Interruptions in power supply to the institutional load may result in loss of crucial data, disrupted academic classes, and equipment damage due to low voltage, among other serious issues. Therefore, a continuous and reliable power supply must be ensured through proper EMS implementation.

Prior studies have focused on the optimal scheduling of microgrid components using various techniques. Problems associated with power outages and power interruptions have also been recognized in previous research. However, electric vehicle scheduling is not widely considered in the context of university campuses. These issues have been addressed separately and there is presently no microgrid optimization tool that considers these issues together. This paper addresses the idea of advancement in the current grid framework at a university campus, focusing on various types of DERs, mainly photovoltaic (PV) system, battery energy storage system (BESS), and electric vehicles (EVs) as prosumers. The concept of an EMS to optimally schedule the power from various energy sources in a grid connected network is proposed. The advantages of the proposed EMS are observed using real data from a university campus as a case study. Grid unavailability in emergency situations is also considered. Currently, the campus load demand is supplied through the local utility. The system used in this study is completely described in Section 3.

The main contributions of this work are as follows:

- An EMS is proposed for optimal scheduling of available energy resources and grid power using robust Linear Programming based on time of use (ToU) pricing scheme to ensure power supply continuity and reduction in energy consumption cost.
- Power outage and power interruption modes are considered for estimating the effect on cost for continuous power supply.
- Effect of integrating EV as a storage device in the proposed microgrid structure is also considered.

The rest of the paper is organized as follows. Section 2 presents the literature review. Section 3 presents the description of the proposed system. Section 4 and Section 5 present the problem formulation. Results and discussion are presented in Section 6. The findings of this paper are concluded in Section 7.

## 2. Literature Review

**Figure 2:**
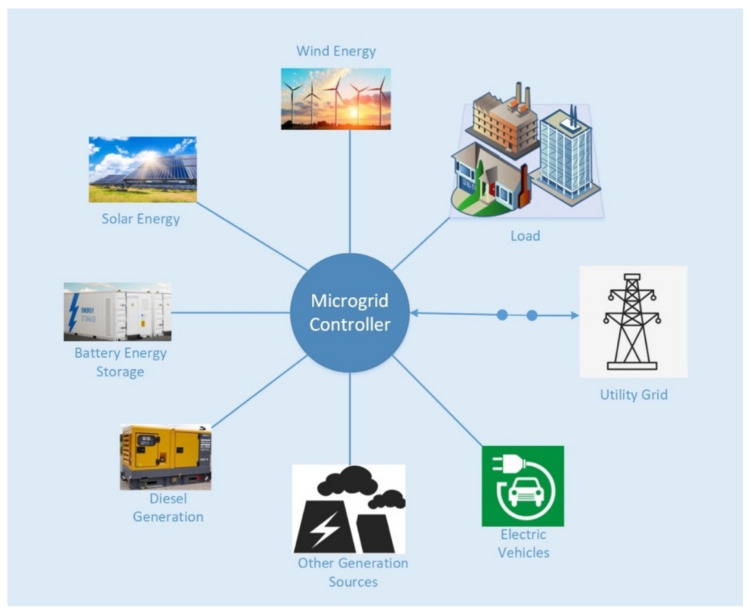
Microgrid system.

Microgrid is a combination of distributed generators (DGs), load and storage system as shown in Figure 2. Microgrids can operate in grid-connected mode or the islanded mode (off-grid mode) [10]. Typically, the islanded mode allows the system to operate in undesirable situations, such as high rates of electricity from main grid, faults in grid, and supplying power to remote areas. In grid-connected mode, the microgrid interacts with the main power grid and can sell or supply surplus energy to the main grid.

The possibility of incorporating usually more than one energy source in the microgrids continues to remain a concern in their planning and analysis.

Microgrid planning has been the subject of numerous research articles. The issue is addressed using mathematical models or software tools and various optimization algorithms. HOMER is a software tool used by many researchers for planning and analysis of microgrids [10,11,12,13,14]. Authors in [12] focused on the feasibility analysis for the development of PV generation plant in one of the campuses of Sebelas Maret University, Indonesia, as a solution for increasing energy demand. The techno-economic analysis was done using HOMER software and was based on NPC (net present cost) and IRR (internal rate of return).

In [13], the authors proposed to develop a grid-connected microgrid including PV and battery storage system to satisfy the load demand and reduce the grid dependency of a campus in Malaysia. HOMER was used to model and simulate the microgrid. The challenges to the introduction of microgrids in the world power sector are described in [14]. The implementation of microgrid technology on a campus in Brazil is considered to illustrate these challenges and to reduce the energy cost. HOMER was used to simulate six arrangements to identify the most techno-economically feasible solution. In [15], a microgrid model comprising combined heat and power plant, diesel generators, PV system and battery storage system was developed to minimize the energy generation cost, greenhouse gas emission, total NPC, and to supply thermal and electrical loads simultaneously. The simulations were carried out using HOMER Pro on six cities in Pakistan to find the best city for the proposed microgrid model.

In [16], the authors proposed a binary backtracking search algorithm to optimally schedule the DGs in microgrids as virtual power plant (VPP). The proposed model was simulated and tested on IEEE 14-bus system based on real data. The results showed a decrease in power losses and cost of generated energy and an increase in reliability. The proposed algorithm gave better results compared to binary particle swarm optimization.

A “Microgrid decision support tool (MDSTool)” for optimal planning of microgrid considering renewable energy incentives, grid ancillary services and tax benefits was developed in [17]. The MDSTool allowed the flexibility to model a wide range of dispatch algorithms. It was a performance and economic model used to find the optimal sizing and financial feasibility of the microgrid. A prototype software architecture was demonstrated in [18] for supporting the data driven demand response (DR) optimization in a campus microgrid. The architecture proposed there has three main components that, when run, displayed the load curtailment patterns in real time and forecasted energy consumption of the campus. In [19], the authors developed a BESS scheduling model for addressing the DR problems. Simulation results showed reduction in operating cost and in DR implementation uncertainty.

The control and monitoring of a functional microgrid at the Illinois Institute of Technology (Chicago, IL, USA) for improving the economics and reliability of microgrids was discussed in [20]. Functions like economical DR, economical dispatch, emergency DR, resynchronization and islanding, self-healing, for implementing microgrid objectives were also discussed. In [21], the authors presented an energy management system (EMS) to integrate PV and BESS in a campus for reducing the energy costs. “Simulated annealing algorithm” was used for implementing the methodology. Simulations were carried out using meteorological data for predicting PV output, real demand, and ToU tariff.

A DSM approach that considered the non-controllable loads triggered by sub-decision makers was proposed in [22]. A micro market model was presented to influence these non-controllable loads. A self-crossover genetic algorithm (SCGA) was proposed to solve the optimization problem. In [23], the authors focused on designing an inertia-based microgrid and its associated controller for the main campus of Clemson University (Clemson, CA, USA). The microgrid could exhibit both on- and off-grid modes and the transition between the two modes was nearly seamless. The idea of development of a microgrid at the Faculty of Technical Sciences at the University of Novi Sad (Novi Sad, Serbia), with various DERs was presented in [24]. That study focused on the technical, economic, and ecological analysis of the microgrid.

In [25], the authors used non-dominated sorting genetic algorithm II (NSGA-II) to optimize the proposed model size comprising PV system, batteries, and grid, in a university in Japan. The authors proposed to use the constant power instead of real-time power. Three scenarios were considered to validate the proposed scheme. It was observed that energy waste was 70 times less if constant power was used throughout the year and 60 times less if it was used daily. The authors in [26] proposed an EMS to analyze the economic and environmental effects of PV and *ESS* in a campus microgrid located in Pakistan. An EMS is proposed in [27] to optimally schedule the PV and storage system in a campus microgrid for reducing energy consumption cost and battery degradation cost. The non-linear problem was formulated using mixed-integer nonlinear programming (MINLP) and simulated in MATLAB.

Considering the previous literature, it can be inferred that most of the work is focused on technical and economic feasibility of the microgrid. Some researchers focused on reducing the grid dependency. PV system and *ESS* integration and management are mostly considered for electricity cost reduction. Moreover, previously proposed studies have rarely focused on EV integration. A comparative analysis of related work is given below in Table 1.

With the introduction of EVs, the issue of catering the increased demand from EVs has become a pressing concern. The management of EVs is currently a serious challenge. This paper considers the effect of application of PV system, *ESS* and EVs as prosumers at a university campus in smart grid. An EMS is proposed to optimally dispatch the energy from available energy resources to ensure continuous power supply and reduce the energy consumption cost.

## 3. System Architecture/Description

The system used in this study is a college on a university campus located in Pakistan. The university main campus encompasses 388.5 ha (960 acres) of land. Currently, it includes over 51 colleges/departments/institutes. The college under study is spread over 28.3 ha (70 acres) of land. It has five departments, a library, a hostel, and residential blocks for staff and faculty.

**Figure 3:**
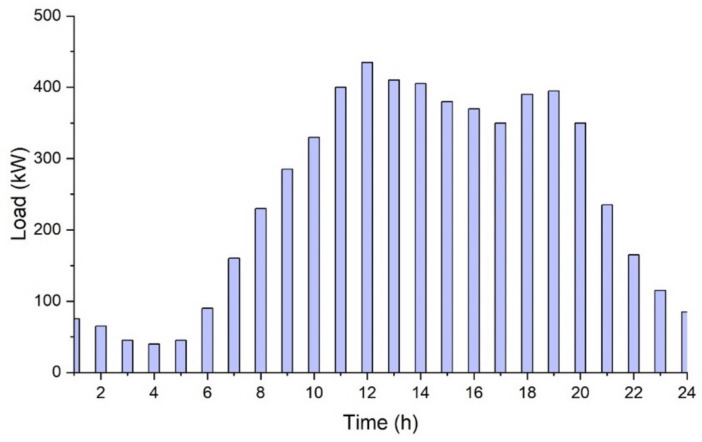
Daily average load profile.

Figure 3 shows the daily average load profile of the College. The primary source of power is the power from the local distribution company named Multan Electric Power Company (MEPCO).

The price of electricity from grid in the time of use (ToU) tariff scheme is shown in Table 2 [28]. Peak hours are from 18:00 to 22:00.

**Figure 4:**
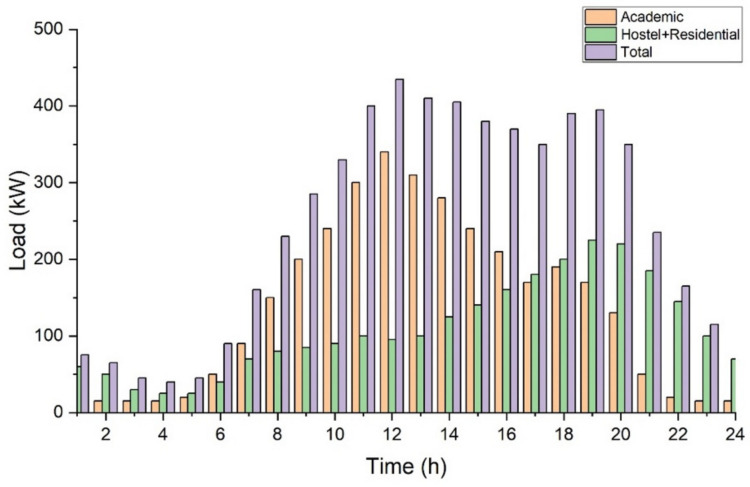
Daily average electrical load distribution pattern.

The total average college electrical load is made up of the average academic, hostel and residential load combined as shown in the Figure 4. The load profile depicts an increase in demand during college timing due to academic load and in the night after college timing due to residential load.

**Figure 5:**
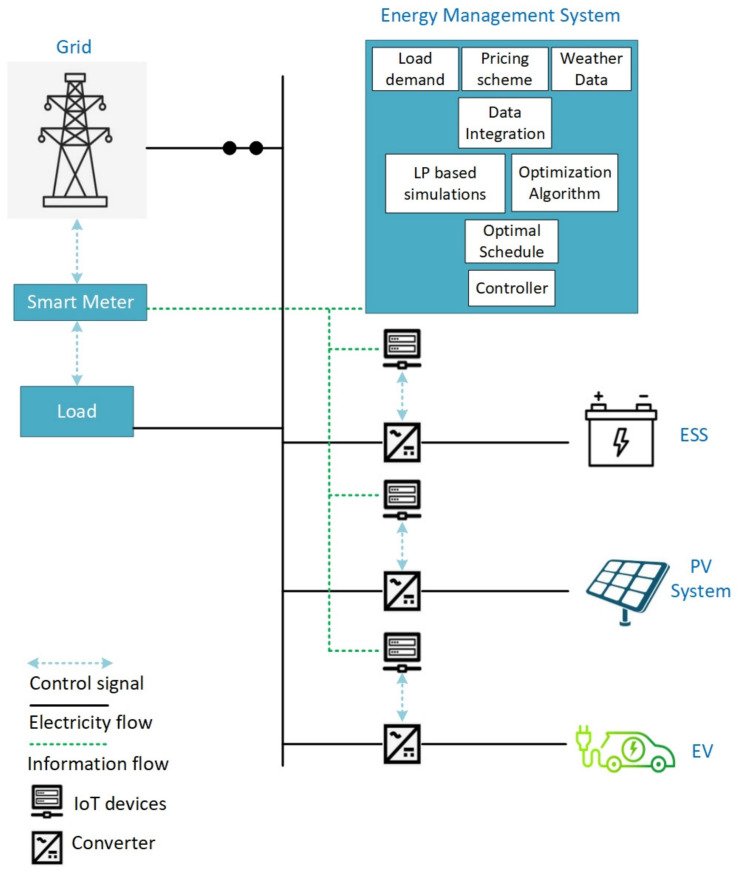
System Architecture.

This paper proposes the integration of a PV system, battery energy storage system (BESS) and electric vehicles (EV) with the distribution system to achieve the energy supply–demand balance and to reduce the electricity consumption cost. Figure 5 shows the proposed system architecture. Following are the details of the proposed system.

### 3.1. Photovoltaic System

**Figure 6:**
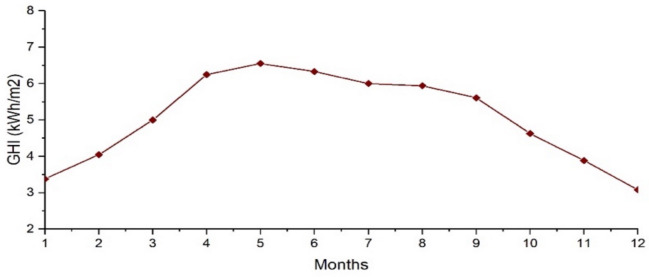
Monthly mean daily GHI of Multan, Pakistan.

Pakistan is one of the world’s fortunate places in terms of solar energy consumption. On a horizontal plane, the average sunlight hours per year are around 1700 to 2200, the average solar radiation range is around 2000 kWh/m^2^/year, 200–250 Watts/m^2^/day, and 2400 kWh/m^2^/year is observed at 30° slope facing South [29,30]. Figure 6 shows the monthly mean daily GHI (global horizontal irradiation) of Multan, Pakistan [31].

In this paper, a total of 400 kW solar PV system is proposed to be built on the building’s rooftops and a solar canopy to be installed at charging station to charge electric vehicles.

### 3.2. Energy Storage System (ESS)

*ESS* technology refers to the process of transforming one form of energy (mostly electrical energy) into another form that can be stored in different mediums. This stored energy can then be transformed back into electrical energy when required [32]. *ESS* are becoming an essential part of smart grid and microgrid infrastructure along with renewable energy sources. An *ESS* helps to overcome the intermittency associated with renewables but also provides the opportunities to apply peak shaving and demand response policies [33,34,35,36]. *ESS* also supports controlling power flow and managing energy storage for grids [37].

BESS [38], flywheel energy storage [39], super capacitors [40], compressed-air energy storage [41], hydrogen energy storage [42], etc. are various types of the *ESS* available. Lithium-ion (Li-ion) batteries are becoming more popular as BESS for their minimal self-discharge rate, better reliability, high power and energy density and extensive lifetime [43]. A Li-ion battery system with 100 kWh of storage capacity is proposed in this paper. The allowable maximum charge and discharge powers are 100 kW and −100 kW respectively. This battery size is selected so that it can store the surplus power generated through PV system and can provide electricity when there are power interruptions or during peak hours.

### 3.3. Electric Vehicle (EV)

Due to increased environmental concerns, many studies are focusing on development of the conventional transport system to AMoD (Autonomous Mobility-on-Demand Systems) [44,45]. Autonomous cars are expected to transform the urban environment, while EVs are already assisting in the transportation sector’s decarbonization.

EVs are an eminent constituent of the modern era, playing a major role in making the road transport greener. However, a large incorporation of EVs in power systems poses new challenges to the power system’s stability and economy attributed to the ample power required for charging their batteries. The deployment of vehicle to grid (V2G) technology can help cope with this issue. In V2G technology, EVs act as storage devices that can store power at off-peak times and can help power the grid at peak times.

**Figure 7:**
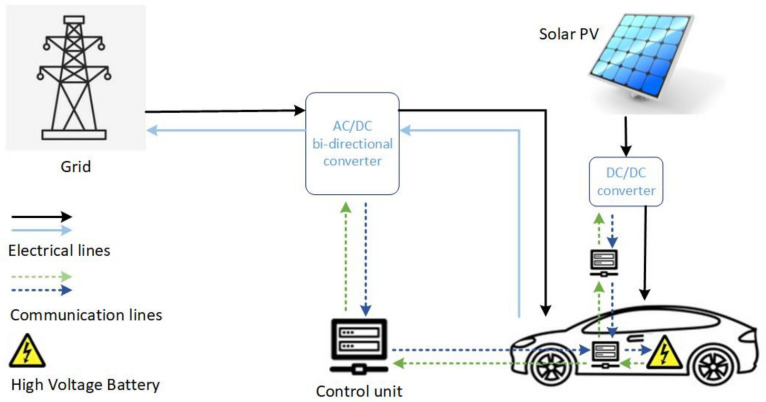
EV integration in the proposed system.

To evaluate the effect of incorporating EVs in the proposed system, it is assumed that the faculty members and the students can park their cars at the charging station during office hours, i.e., 09:00 to 15:00. EVs will get charged through the PV generation and the grid supply. The cars can only charge and discharge while parked. EVs will try to get charged to their maximum capacity. EVs will act as a source to supply power to grid when there is a fault in grid or when there is an instant increase in load demand. Figure 7 shows the integration of EVs in the proposed system [46].

## 4. Mathematical Modelling

### 4.1. PV System Modelling

PV modules power output generally depends on solar irradiance and temperature, and can be calculated as follows [21]:

(1) $$P_{PV}=N\eta_{inv}\left[P_{n,r}\frac{G_{k}}{G_{k,r}}\left(1+\gamma \left(T_{c}-T_{r}\right)\right)\right],$$

where $P_{PV}$ is the PV output power, *n* is the total number of modules in the PV system, $\eta_{inv}$ is the efficiency of inverter, $P_{n,r}$ is the nominal power under reference test conditions for each module, $G_{k}$ is the solar irradiance at time instant t, $G_{k,r}$ is the solar irradiance under reference test conditions, γ is the temperature coefficient, $T_{c}$ and $T_{r}$ represent the cell temperature and cell temperature under reference test conditions, respectively. The model is further explained in the relationship:

(2) $$T_{c}=T_{amb}+\left(\frac{N_{o}-20}{0.8}\right)G_{k},$$

where $N_{o}$ is the “Nominal Operating Cell Temperature” and $T_{amb}$ is the ambient temperature. The installed PV capacity, $P_{C}$, can be determined using the following equation [12]:

(3) $$P_{C}=\eta_{pv}.A.\omega_{p},$$

where $\eta_{pv}$ is the efficiency of PV, A is the area of solar panel and $\omega_{p}$ is the peak solar insulation. PV operating costs are considered to be a fixed cost of maintenance per time interval. The DC power output of the PV system is converted to AC power through the application of an inverter. The solar modules power $P_{A}$ and typical efficiency of the inverter are used to estimate the rating of the inverter, according to the following equation:

(4) $$C_{inv}=P_{A}\frac{100}{\eta_{inv}}$$

The solar data used in this paper are taken from [26].

### 4.2. Energy Storage System Constraints

The binary variable for charging and discharging state of battery are $X_{ESS}^{c}$ and $X_{ESS}^{d}$,

(5) $$X_{ESS}^{c},X_{ESS}^{d}\in \left\{0,1\right\}$$

Charging and discharging power level constraints are given in the equations below. It shows that the charging power will be zero if $X_{ESS}^{c}=0$, that is, *ESS* is not in the charging mode, and the same holds true for discharging mode if $X_{ESS}^{d}=0$.

(6) $$P_{min}^{c}\leq P_{t}^{c}\leq X_{ESS}^{c}P_{max}^{c},$$

(7) $$P_{min}^{d}\leq P_{t}^{d}\leq X_{ESS}^{d}P_{max}^{d},$$

where $P^{c}$ and $P^{d}$ are the charging and discharging powers respectively.

At each time instant, the battery must either be in charging or discharging state.

(8) $$X_{ESS}^{c}+X_{ESS}^{d}\leq 1$$

Fully discharging the battery can damage the system. For that reason, the charging and discharging of *ESS* must be within the limits specified by the following equations [27]:

(9) $$\frac{SOC_{t-1}-SOC_{max}}{100}C_{ESS}\leq P_{ESS,t},$$

(10) $$\frac{SOC_{t-1}-SOC_{min}}{100}C_{ESS}\geq P_{ESS,t},$$

where *SOC* is the state of charge of battery, and $P_{ESS}$ is the power output of the battery.

The $SOC_{t}$ level of battery at time instant t depends on the previous state $\left(SOC_{t-1}\right)$, and can be calculated using the equation given below:

(11) $$SOC_{t}=SOC_{t-1}-\frac{P_{ESS,t}\times 100}{C_{ESS}}$$

To avoid accelerated aging of the battery, the following equation must be satisfied

(12) $$SOC_{min}\leq SOC_{t}\leq SOC_{max}$$

The *SOC* level should be the same at the end and start of an optimization period as *ESS* is operated daily.

(13) $$SOC_{\left(t_{o}\right)}=SOC_{\left(t_{end}\right)}$$

### 4.3. Electric Vehicle Constraints

EVs act as storage tools, provided that they are connected to a charging station. The *SOC* for the kth EV $\left(SOC_{k}\right)$, between the arrival and departure time, can be evaluated using the equation given below [47]:

(14) $$SOC_{k,t}=SOC_{k,t-1}+\left[\eta_{EV}^{c}P_{k,t}^{c}-\frac{P_{k,t}^{d}}{\eta_{EV}^{d}}\right]$$

The power demand of the kth EV at time t, $P_{k,t}$, can be calculated from the following equation [48]:

(15) $$P_{k,t}=P_{k}s_{t}\omega_{t}h_{t},$$

where, $P_{k}$ is the rated power of the kth EV, $s_{t}$ represents the connectivity status of EV in time t, $\omega_{t}$ denotes the weekdays and $h_{t}$ denotes the working hours. For various EVs, the aggregated EV power demand is calculated by adding power demand of each EV.

(16) $$P_{EV,t}=\left\{\begin{array}{cc} \sum_{k=1}^{N}P_{k,t}, & \left(time\;to\;charge\;EVs\right)\\ 0, & else \end{array}\right.$$

The charging and discharging power level constraints are considered to ensure that the charging and discharging power levels at each time interval are within the limits.

(17) $$P_{k,min}^{c}\leq P_{k,t}^{c}\leq P_{k,max}^{c},$$

(18) $$P_{k,min}^{d}\leq P_{k,t}^{d}\leq P_{k,max}^{d}$$

The *SOC* for the kth EV at time t, must be within limits to prevent the impact on state of health (SOH) of the battery.

(19) $$SOC_{k,t}^{min}\leq SOC_{k,t}\leq SOC_{k,t}^{max}$$

### 4.4. Grid Connection

The connection between the grid and microgrid has a maximum power exchange limit, implying that the power purchased from the grid or the power sold to the grid should be within the limits as given below [49],

(20) $$-P_{Grid}^{min}\leq P_{Grid}\leq P_{Grid}^{max}$$

To achieve the supply–demand balance, the following equation must be satisfied.

(21) $$P_{Grid,t}+P_{PV,t}+P_{ESS,t}+P_{EV,t}=P_{Load,t}$$

## 5. Objective Function

The objective problem is formulated as a linear optimization problem to minimize the daily electricity consumption cost by optimally scheduling the power sources as given below,

(22) $$min\;J=\sum_{t=1}^{T}\left[J_{Grid,t}+J_{PV,t}+J_{ESS,t}+J_{EV,t}\right],$$

where,

(23) $$J_{Grid,t}=(P_{Grid,t})R_{Grid,t},$$

(24) $$J_{PV,t}=(P_{PV,t})R_{PV,t},$$

(25) $$J_{ESS,t}=(P_{ESS,t})R_{ESS,t},$$

(26) $$J_{EV,t}=(P_{EV,t})R_{EV,t}$$

In Equation (22)–(26), *J* represents the electricity cost, $J_{Grid,t}$, $J_{PV,t}$, $J_{ESS,t}$ and $J_{EV,t}$ are the electricity costs at time t, associated with grid, photo-voltaic system, energy storage system and electric vehicles, respectively. The unit price at any time is represented by $R_{t}$.

### Solution Methodology

The optimization problem is solved using robust linear programming. It is a technique that considers linear relationships to find the optimal solution for the given problem. It reaches towards the global optima using less computation time and is seldom used at campus microgrid sites. The general expression of linear programming is as follows:

(27) $$\underset{x}{min}\;f^{T}x,$$

such that

(28) $$\left\{\begin{array}{c} A.x\leq b,\\ A_{eq}.x=b_{eq},\\ ub\leq x\leq lb. \end{array}\right.$$

In Equations (27) and (28), *f*, *x*, *b*, *beq*, *lb*, and *ub* are vectors, and *A* and *Aeq* are matrices.

Interior-point method is used in MATLAB to solve the objective function. Internet of things (IoT) based devices are used for monitoring and control [50,51]. IoT plays an essential role in connecting devices, processes, and things. It considerably improves communication features and provides latest data to the distributed networks.

## 6. Results and Discussion

Following cases are analyzed using Linear programming in MATLAB to achieve the best outcome. These cases differ with respect to the components and parameters that are used in them. Comparing the cases with a reference or base case is important for microgrid analysis and planning. The reference case is how the system is currently operating prior to microgrid implementation. The reference case and the other cases compared to the reference case are described in this section.

Table 3 lists the components and parameters used in each case.

### 6.1. Case 1, Grid Only

No PV, *ESS* and EV are considered in this case. Power is solely supplied by the utility grid. The electricity cost using ToU tariff in this case is evaluated to be $622.42 per day. This case is considered as a reference case. It indicates how the system is operating currently.

### 6.2. Case 2, Grid with PV and ESS

**Figure 8:**
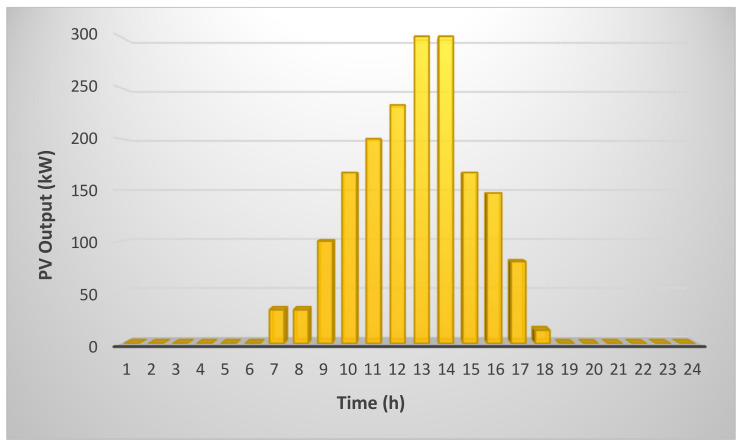
PV production on an average day during the year.

In this case, the effect of implementation of PV and *ESS* are considered along with grid. PV production on an average day during a year is shown in Figure 8 [26]. It can be observed in the figure that from night until 7:00 in morning, there is little or no sunlight to produce power. After this, PV captures sunlight to produce power, reaching its maximum at noon.

**Figure 9:**
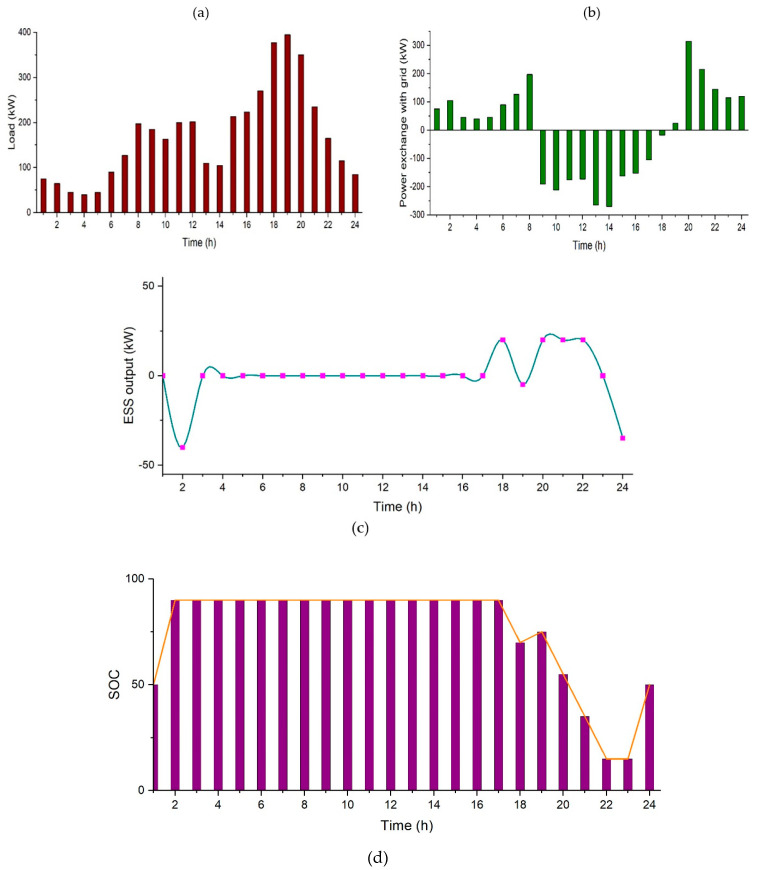
Case 2, (**a**) College prosumer load profile, (**b**) Power exchange with the grid, (**c**) Power output of *ESS*, and (**d**) State of charge of the battery.

The daily average load profile of the college is shown in Figure 3, which is the normal load profile in the absence of any generation or storage by the microgrid. However, after setting up the PV system with *ESS*, the load profile changes entirely. This is because when we have a PV system coupled with some load, the final load profile is a result of the difference between the original load profile and PV production. This is clearly shown in Figure 9b, where the load has been fairly reduced, and the reduction corresponds to the production from the PV system.

The total electricity cost comes out to be $343.64 over a day. There is a reduction of nearly 45% from the original cost considering only grid. Figure 9 shows the simulation results for this case, where Figure 9a represents the college prosumer load profile, Figure 9b shows the power exchange with the grid, Figure 9c represents the power output of *ESS*, and Figure 9d shows the state of charge of the battery.

### 6.3. Case 3, Grid with PV and *ESS* Considering Power Interruptions

**Figure 10:**
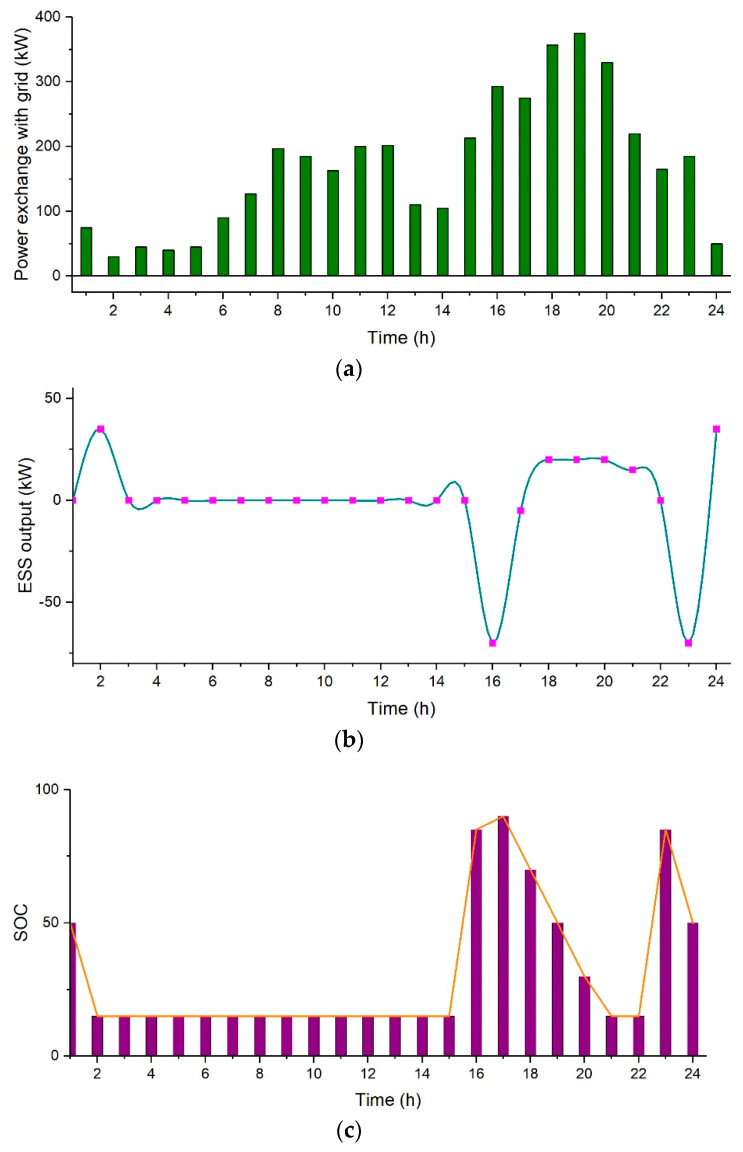
Case 3, (**a**) Power exchange with the grid, (**b**) Power output of *ESS*, and (**c**) State of charge of battery.

There are situations when the main grid is not available to support the load demand such as during power outages. In these situations, the microgrid is on its own, i.e., the microgrid is operating in off-grid mode. In this case, the effect of power outages is evaluated along with PV, *ESS* and grid. It is assumed that there is shortage of power from 11:00 to 12:00 and 17:00 to 18:00. At that time, the power will be supplied through *ESS* and PV as depicted through the simulation results. PV production is near the maximum from 11:00 to 12:00, so the batteries stay idle, and the load is fed directly by the PV system. During hours with low PV production, such as from 17:00 to 18:00, the load is being fed by the batteries instead, which can be clearly seen in the *SOC* curve where the batteries are in a discharging state from 17:00–18:00 h. The total cost in this case comes out to be $461.99 per day. In Figure 10, Figure 10a shows power exchange with the grid, Figure 10b represents the power output of *ESS*, and Figure 10c represents the state of charge of battery.

### 6.4. Case 4, Grid with PV, ESS, and EV

In this case, the effect of integrating EVs is estimated. A total of 100 EVs are assumed for the case study. Two scenarios are considered, (I): EVs are acting as a load, and (II): EVs are acting as a source.

#### 6.4.1. EV as a Load

**Figure 11:**
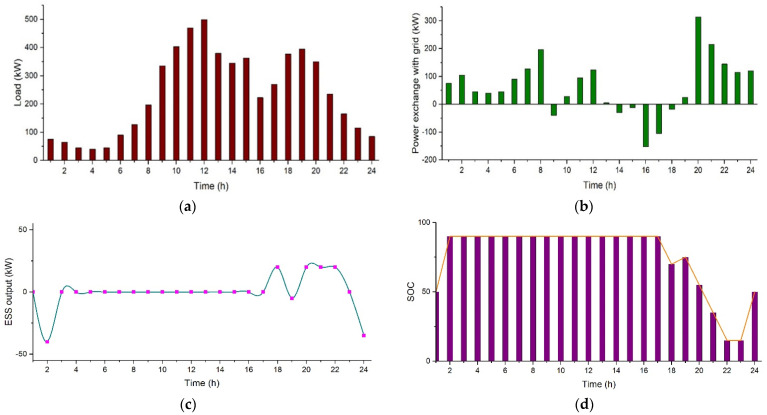
Case 4 (I), (**a**) College prosumer load profile with EVs integration, (**b**) Power exchange with the grid, (**c**) Power output of *ESS*, and (**d**) State of charge of battery.

In this scenario, EVs are acting as a load. The average load profile with EVs integrated in the system is shown in Figure 11a [52]. EVs will try to get charged to their maximum capacity. EVs can store energy while acting as a load and can supply this stored energy to the grid during peak times. Simulation results show that the total electricity cost for this case is $502.10 per day. Figure 11 b depicts that less power is exported to the grid in this case from 09:00 to 15:00, as most of the power is used to charge the EVs during that time. In Figure 11, Figure 11a represents the college prosumer load profile with EVs integration, Figure 11b shows power exchange with the grid, Figure 11c represents the power output of ESS, and Figure 11d represents the state of charge of battery.

#### 6.4.2. EV as a Source

**Figure 12:**
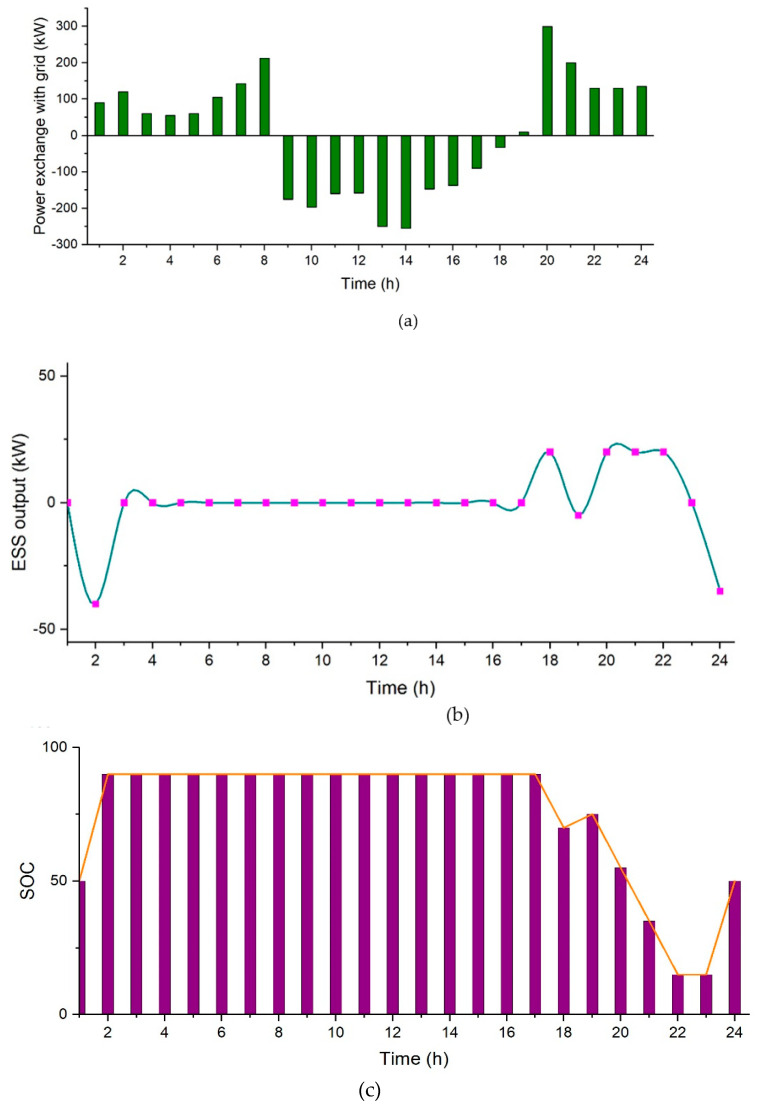
Case 4 (II), (**a**) Power exchange with the grid, (**b**) Power output of *ESS*, and (**c**) State of charge of battery.

In this case, EVs act as a source of electricity by supplying the stored energy to the loads and grid. It is assumed that the EVs act as a storage system of 100 kWh. Simulation results show that the total electricity cost for this case is $338.72 per day. In Figure 12, Figure 12a shows power exchange with the grid, Figure 12b represents the power output of *ESS*, and Figure 12c represents the state of charge of battery. It can be seen in Figure 12a that a large amount of power is exported to the grid, in this case with an added storage of 100 kWh.

Integrating DERs with existing grid and optimal scheduling of available energy resources is analyzed in the above-mentioned cases. The electricity cost and cost saving associated with each case is summarized in Table 4. The cost is highest in the reference case without any DERs. Integrating PV and *ESS* with the grid has decreased the cost by nearly 45%. Of note, in [26], the cost saving was 35% with PV, *ESS*, and diesel generator, using mixed-integer linear programming. EVs were not considered in this work.

Integrating EVs as source has reduced the cost by 45.58% and as load by 19.33%. In case of power interruptions, the EMS optimally utilizes the energy reserves to ensure continuous power supply and the cost is reduced by 25.78%. Thus, a microgrid scheduler is required for seamless and cost-effective operation. The last column shows the levelized cost of energy (LCOE) ($/kWh), which is based on the installation, operation, and maintenance costs.

## 7. Conclusions

In this paper, the effect of incorporating PV system, *ESS* and EVs are studied in a distribution network of a university campus. The study proposes the application of an optimal EMS to the system considering the future connections of the proposed DERs. The problem was formulated using the linear optimization technique and simulated in MATLAB. The impact of the proposed EMS was observed considering various cases. The energy requirement of the college was solely supplied through the local utility under the time of use (ToU) tariff without any DERs, resulting in high energy consumption cost. Integrating PV and *ESS* in the system implied a cost saving of 44.80% daily. The impact of real-time local problems like power outages was also investigated. The proposed EMS ensured the continuous power supply to the load during the grid outage hours without compromising the daily activities at the college. Moreover, the effect of integrating EVs in the current infrastructure was also studied. The impact of EV as a load and as an energy source were investigated discretely. Providing the load demand presented by EVs resulted in a daily percentage saving of 19.33% and the savings increased to 45.58% when EVs were used as a source to provide for the load. The future work will involve expanding the project to encompass the whole main campus. The uncertainties associated with DERs and complex mathematical models will be researched in the future.

## Figures and Tables

**Table 1:** Comparative study of related work.

Ref	[12]	[13]	[14]	[17]	[18]	[20]	[21]	[22]	[23]	[24]	[26]
**Components**	PV, BESS	PV, BESS	PV, Wind	PV, ESS, Converter		PV, wind, BESS, natural-gas turbine generator	PV, BESS		PV, BESS, Inverters, Diesel generator	PV, Wind, EV, BESS, CHP power plant	PV, BESS, Diesel generator
**Algorithms**				Dispatch Algorithm	Machine-learning techniques, complex event processing (CEP)		Simulated Annealing	self-crossover genetic algorithm			Mixed-integer linear programming
**Software**	HOMER	HOMER	HOMER	MATLAB	Semantic database	SCADA	MATLAB	MATLAB Toolbox	PSCAD		MATLAB
**Campus Name**	Sebelas Maret University, Indonesia	University of Kuala Lumpur, Malaysia	Federal University of Rio de Janeiro, Brazil	Seoul National University, South Korea	University of Southern California (USC), Los Angeles	Illinois Institute of Technology (IIT), Chicago	Federal University of Para, Brazil	Anonymous	Clemson University, South Carolina	University of Novi Sad, Serbia	U.E.T, Taxila, Pakistan
**Validity**	The results were analyzed based on NPC and IRR methods	Based on total net present cost	Comparison between six different technical arrangements	Comparison without microgrid	The portal will display real-time load curtailment patterns that are detected by the CEP system for these buildings	Permanent 20% decrease in the peak load from the 2007 level	Three cases, Reference, PV, PV and BESS, are compared	Comparison with traditional optimization algorithms	The system satisfies IEEE Std 1547.4	The microgrid is analyzed on technical, economic and ecological basis.	The microgrid is analyzed on economic and environmental basis
**Objectives**	Minimize the net present and operating costs of the system	Meet the campus load demand and minimize grid dependency	Minimize energy costs	Minimize total operating cost	Data driven DR optimization	Enhancing the microgrid reliability and economics	Minimize campus energy consumption cost	Minimize overall energy cost of the system		GHG emission reduction	Energy cost and GHG emission reduction
**Constraints**			Budgetary constraints	Power balance, operational, ramp up/down			State of charge and Power constraints of BESS	Equality and inequality constraints	SOC constraints		ESS constraints
**Voltage/System level**	Large office building	British Malaysian Institute	Technology Center	Three buildings (selected)	Three buildings on campus		13.8-kV	Education and research	12.5 kV	Faculty of Technical Sciences	University campus

**Table 2:** Electricity prices.

Time (h)		Price ($/kWh)
1:00–18:00	Off peak	0.098
18:00–22:00	Peak	0.13
22:00–24:00	Off peak	0.098

**Table 3:** Case studies profile.

Components & Parameters	Case 1 (Reference Case)	Case 2	Case 3	Case 4
Grid	√	√	√	√
PV	×	√	√	√
BESS	×	√	√	√
EV	×	×	×	√
Power interruptions	×	×	√	×

**Table 4:** Comparison of electricity cost for each case.

Cases	Cost ($/Day)	Cost Saving (%)	LCOE ($/kWh)
1 (Reference case)	622.42	0	0.097
2	343.64	44.80	0.053
3	461.99	25.78	0.072
4 (I)	502.10	19.33	0.078
4 (II)	338.72	45.58	0.052

## Data Availability

Not Applicable.
